# Supplementary material for: Biodegradation of Crystal Violet dye by bacteria isolated from textile industry effluents
Source: PeerJ. 2018 Jun 21;6:e5015. doi: 10.7717/peerj.5015 (PMC6015751; doi:10.7717/peerj.5015)
Supplement: Supplemental Information 1 [file peerj-06-5015-s001.docx]

**Table : Characteristics of collected samples**

| **Sample** | **Nature of sample** | **Color** | **Temperature (°C)** |
| --- | --- | --- | --- |
| **Water 1** | Liquid | Black | 18 |
| **Sludge 1** | Muddy | Black | 18 |
| **Water 2** | Liquid | Turquoise blue | 18 |
| **Sludge 2** | Muddy | Black | 18 |
