# Supplementary material for: Biodegradation of Crystal Violet dye by bacteria isolated from textile industry effluents
Source: PeerJ. 2018 Jun 21;6:e5015. doi: 10.7717/peerj.5015 (PMC6015751; doi:10.7717/peerj.5015)
Supplement: Supplemental Information 2 [file peerj-06-5015-s002.docx]

**Table :** Effect of temperature on crystal violet dye degradation by *Enterobacter* sp. CV–S1

| **Temperature** | **Initial OD** | **Final OD** | **Degradation rate (%)** | **Average degradation rate (%)** | **Duration of observation** |
| --- | --- | --- | --- | --- | --- |
|  | 0.04 | 0.025 | 37.5 |  |  |
| 30°C | 0.04 | 0.025 | 37.5 | 37.5 | 24 hours |
|  | 0.04 | 0.025 | 37.5 |  |  |
|  | 0.04 | 0.00 | 100 |  |  |
| 35°C | 0.04 | 0.00 | 100 | 100 | 24 hours |
|  | 0.04 | 0.00 | 100 |  |  |
|  | 0.04 | 0.025 | 37.5 |  |  |
| 40°C | 0.04 | 0.025 | 37.5 | 37.5 | 24 hours |
|  | 0.04 | 0.025 | 37.5 |  |  |
