# Supplementary material for: Biodegradation of Crystal Violet dye by bacteria isolated from textile industry effluents
Source: PeerJ. 2018 Jun 21;6:e5015. doi: 10.7717/peerj.5015 (PMC6015751; doi:10.7717/peerj.5015)
Supplement: Supplemental Information 3 [file peerj-06-5015-s003.docx]

**Table :** Effect of pH on crystal violet dye decolorization by *Enterobacter* sp. CV–S1

| **pH** | **Initial OD** | **Final OD** | **Degradation rate (%)** | **Average Degradation rate (%)** | **Duration of observation** |
| --- | --- | --- | --- | --- | --- |
|  | 0.04 | 0.035 | 12.5 |  |  |
| 6.00 | 0.04 | 0.035 | 12.5 | 12.5 | 24 hours |
|  | 0.04 | 0.035 | 12.5 |  |  |
|  | 0.04 | 0.00 | 100 |  |  |
| 6.50 | 0.04 | 0.00 | 100 | 100 | 24 hours |
|  | 0.04 | 0.00 | 100 |  |  |
|  | 0.04 | 0.005 | 87.5 |  |  |
| 7.00 | 0.04 | 0.005 | 87.5 | 87.5 | 24 hours |
|  | 0.04 | 0.005 | 87.5 |  |  |
|  | 0.04 | 0.025 | 37.5 |  |  |
| 7.50 | 0.04 | 0.025 | 37.5 | 37.5 | 24 hours |
|  | 0.04 | 0.025 | 37.5 |  |  |
|  | 0.04 | 0.03 | 25.00 |  |  |
| 8.00 | 0.04 | 0.03 | 25.00 | 25.00 | 24 hours |
|  | 0.04 | 0.03 | 25.00 |  |  |
|  | 0.04 | 0.03 | 25.00 |  |  |
| 8.50 | 0.04 | 0.03 | 25.00 | 25.00 | 24 hours |
|  | 0.04 | 0.03 | 25.00 |  |  |
