# Supplementary material for: Biodegradation of Crystal Violet dye by bacteria isolated from textile industry effluents
Source: PeerJ. 2018 Jun 21;6:e5015. doi: 10.7717/peerj.5015 (PMC6015751; doi:10.7717/peerj.5015)
Supplement: Supplemental Information 4 [file peerj-06-5015-s004.docx]

**Table :** Effect of inoculum size on crystal violet dye degradation by *Enterobacter* sp. CV–S1

| **Inoculum size [(%) v/v]** | **Initial OD** | **Final OD** | **Degradation rate (%)** | **Average Degradation rate (%)** | **Duration of observation** |
| --- | --- | --- | --- | --- | --- |
|  | 0.12 | 0.01 | 91.67 |  |  |
| 8 | 0.12 | 0.01 | 91.67 | 91.67 | 72 hours |
|  | 0.12 | 0.01 | 91.67 |  |  |
|  | 0.12 | 0.005 | 95.83 |  |  |
| 9 | 0.12 | 0.005 | 95.83 | 95.83 | 72 hours |
|  | 0.12 | 0.005 | 95.83 |  |  |
|  | 0.12 | 0.00 | 100 |  |  |
| 10 | 0.12 | 0.00 | 100 | 100 | 72 hours |
|  | 0.12 | 0.00 | 100 |  |  |
