# Supplementary material for: Biodegradation of Crystal Violet dye by bacteria isolated from textile industry effluents
Source: PeerJ. 2018 Jun 21;6:e5015. doi: 10.7717/peerj.5015 (PMC6015751; doi:10.7717/peerj.5015)
Supplement: Supplemental Information 5 [file peerj-06-5015-s005.docx]

**Table :** Effect of initial crystal violet dye concentration on degradation by *Enterobacter* sp. CV–S1

| **Dye concentration ( mg/l)** | **Initial OD** | **Final OD** | **Degradation rate (%)** | **Average Degradation rate (%)** | **Duration of observation** |
| --- | --- | --- | --- | --- | --- |
|  | 0.04 | 0.00 | 100 |  |  |
| 50 | 0.04 | 0.00 | 100 | 100 | 24 hours |
|  | 0.04 | 0.00 | 100 |  |  |
|  | 0.08 | 0.00 | 100 |  |  |
| 100 | 0.08 | 0.00 | 100 | 100 | 48 hours |
|  | 0.08 | 0.00 | 100 |  |  |
|  | 0.12 | 0.00 | 100 |  |  |
| 150 | 0.12 | 0.00 | 100 | 100 | 72 hours |
|  | 0.12 | 0.00 | 100 |  |  |
|  | 0.16 | 0.09 | 43.75 |  |  |
| 200 | 0.16 | 0.09 | 43.75 | 43.75 | 72 hours |
|  | 0.16 | 0.09 | 43.75 |  |  |
