# Supplementary material for: Biodegradation of Crystal Violet dye by bacteria isolated from textile industry effluents
Source: PeerJ. 2018 Jun 21;6:e5015. doi: 10.7717/peerj.5015 (PMC6015751; doi:10.7717/peerj.5015)
Supplement: Supplemental Information 6 [file peerj-06-5015-s006.docx]

**RESULTS AND DISCUSSION**

The treatment of textile effluent containing dye has been carried out by various physical and chemical methods over the last two decades for the removal of color from textile waste water (Bagewadi *et al*., 2011). These methods are generally expensive, produce large amounts of sludge. More often these conventional modes of treatment lead to the formation of some harmful side products (Tripathi and Srivastava, 2011). New processes for dye degradation and wastewater treatment and reutilization are being developed, (Santos *et al*., 2007). In particular, systems based on biological processes using a large variety of bacterial strains, allow for degradation and mineralization with a low environmental impact and without the use of potentially toxic chemical substances, under mild pH and temperature conditions (Dhanve *et al*., 2008; Khalid *et al*., 2008).

Thus, the present investigation was based on isolation, screening and identification of textile dye degrading bacteria and optimization of environmental parameters on dye degradation by the screened *viz.* identified bacteria.

**4.1** **Isolation, Screening and Identification of Dye Degrading Bacteria**

**4.1.1 Physical Characteristics of Collected Samples**

Physical characteristics of collected samples from Kumarkhali (under Kushtia district, Bangladesh) were shown in Table 4.1. The samples were collected in sterilized container from respective sites. The color, temperature and pH of the sample were recorded on the site and samples were transported to the laboratory.

**Table 4.1:** Characteristics of collected samples

| **Sample** | **Nature of sample** | **Color** | **Temperature (°C)** |
| --- | --- | --- | --- |
| **Water 1** | Liquid | Black | 18 |
| **Sludge 1** | Muddy | Black | 18 |
| **Water 2** | Liquid | Turquoise blue | 18 |
| **Sludge 2** | Muddy | Black | 18 |

**4.1.2 Isolation of Textile Dye degrading Bacteria**

Isolation of textile dye decolorizing bacteria from textile effluent is shown in Figure 4.1. The selective enrichment of sludge 2 [Figure 4.1(A)–(B)] sample among four led to the isolation of 6 two triphenylmethane dye degrading bacterial isolates based on colony characteristics [Figure 4.1(C)–(D)]. The isolates from CV sludge 2 plate were named as CV–S1, CV–S2 and CV–S3 for crystal violet dye and, from CM sludge 2 plate named as CM–S1, CM–S2 and CM–S3 for mixed (crystal violet and malachite green) dye degradation. After 24 hours culturing isolated distinct colonies showed dye degradation when inoculated in enrichment medium [Figure 4.1(E)–(F)].

**4.1.3 Colony Characteristics and Microscopic Morphology of the Isolated Bacteria**

Colony characteristics and microscopic morphology of the isolated bacteria are presented in Table 4.2 and Figure 4.2. When isolated bacteria was grown on nutrient agar plate, there were various characteristics like pigmentation, size, shape, texture, consistency, opacity and so on were found as shown in Table 4.2 and Figure 4.2(A). The Gram’s staining shown in Figure 4.2(B) indicates that out of 6; 3 isolates were Gram negative cocci and rest 3 isolates were Gram negative rod.

**Table 4.2:** Colony characteristics of dye (crystal violet and crystal violet and malachite green) decolorizing bacterial isolates on Nutrient agar

| **Isolate** | **Color** | **Shape** | **Size** | **Opacity** | **Elevation** | **Surface texture** | **Stickiness** |
| --- | --- | --- | --- | --- | --- | --- | --- |
| CV – S1 | White | Irregular | Large | Opaque | Flat | Rough | Yes |
| CV – S2 | White | Irregular | Large | Translucent | Flat | Rough | No |
| CV – S3 | White | Round | Large | Opaque | Convex | Smooth | No |
| CM – S1 | White | Irregular | Large | Opaque | Flat | Rough | Yes |
| CM – S2 | White | Round | Large | Opaque | Convex | Smooth | Yes |
| CM – S3 | Reddish | Irregular | Large | Translucent | Flat | Rough | No |


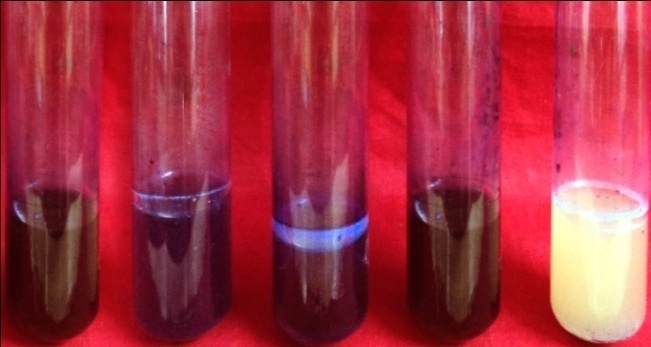

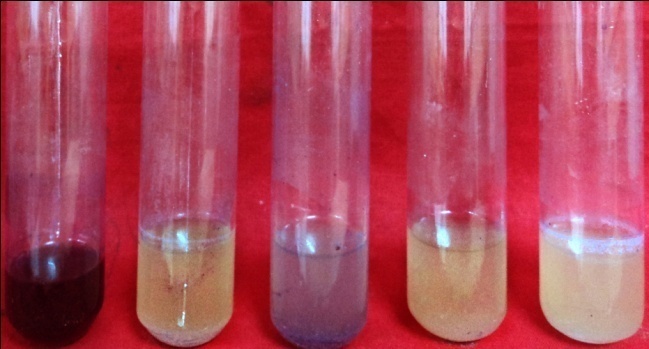


**Control**

**E**

**F**

**Control**

**CV–S1**

**CV–S2**

**CV–S3**

**CM–S1**

**CM–S3**

**CM–S2**

**C**

**CV sludge 2**

**CM sludge 2**

**D**

**Control**

**Water 1**

**Water 2**

**Sludge 1**

**Sludge 2**

**A**

**B**

**Control**

**Water 1**

**Water 2**

**Sludge 1**

**Sludge 2**


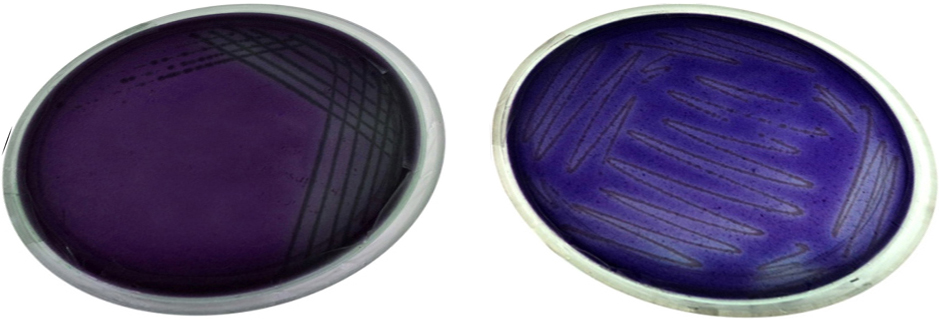


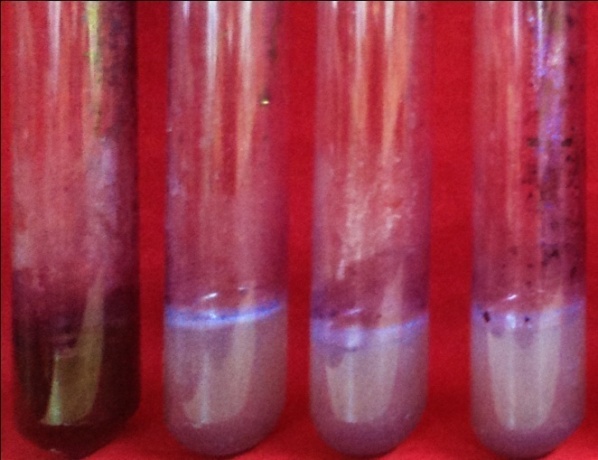

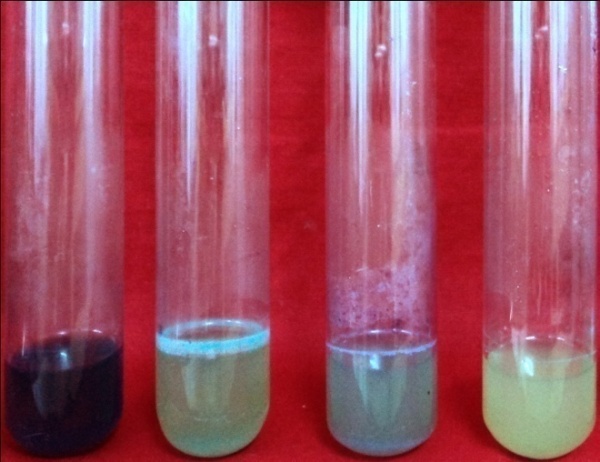


**Figure 4.1:** Isolation of dye degrading bacteria: Selection of highly potential sample: different samples in enrichment medium containing (**A)** Crystal violet and (**B)** Mixed (crystal violet and malachite green) dye; Streak plating from most decolorized sample (sludge 2) on enrichment agar medium containing (**C**) Crystal violet dye and (**D)** Mixed (crystal violet and malachite green) dye to isolate potential bacteria from the most decolorized sample; Efficiency determination of the isolated bacteria in enrichment medium containing (**E)** Crystal violet (**F)** Mixed (crystal violet and malachite green) dye

**
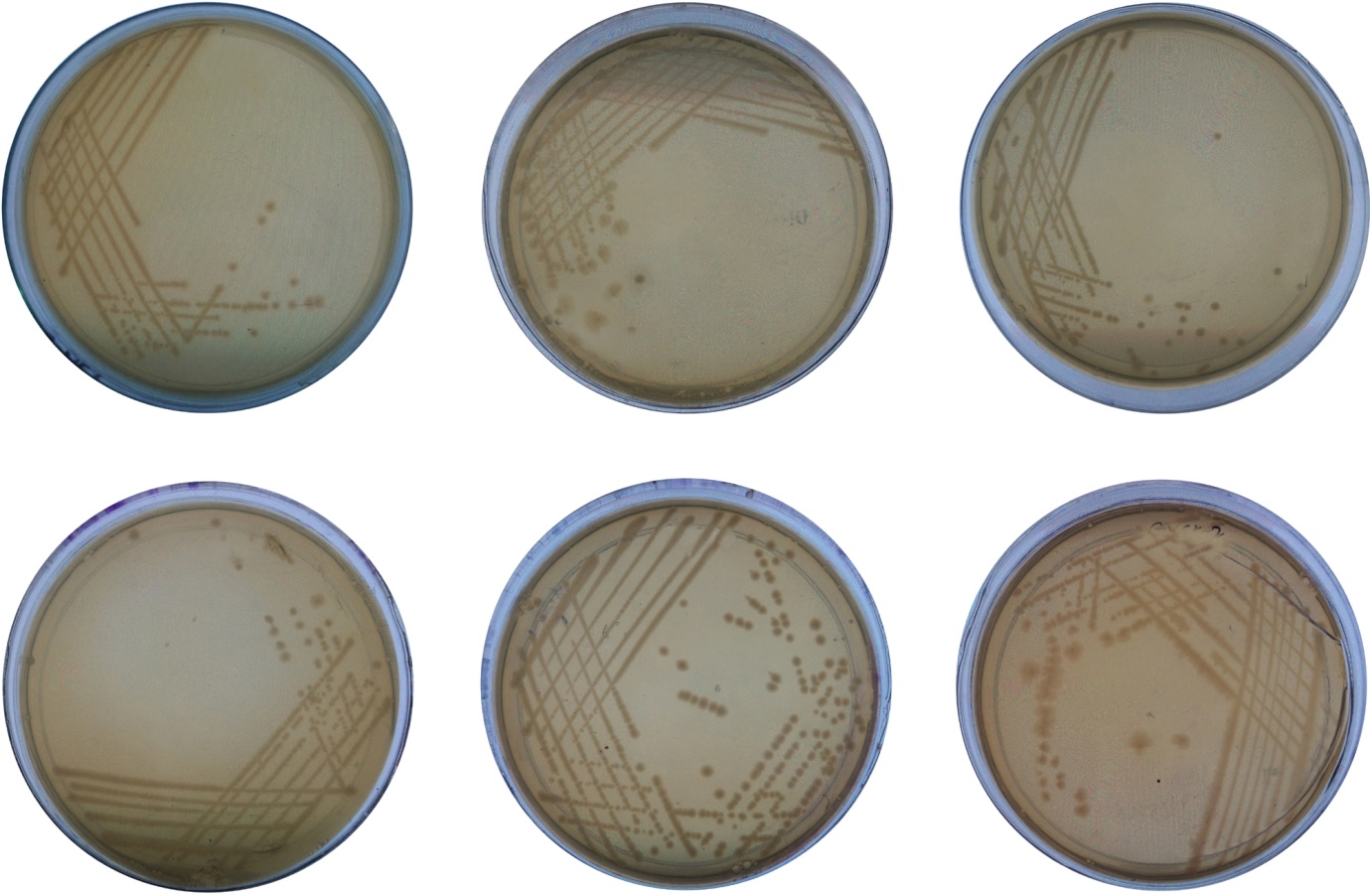
**

**CV–S1**

**CV–S2**

**CV–S3**

**CM–S1**

**CM–S3**

**CM–S2**

**A**

**B**

**CV–S1**

**CV–S2**

**CV–S3**

**CM–S1**

**CM–S2**

**CM–S3**


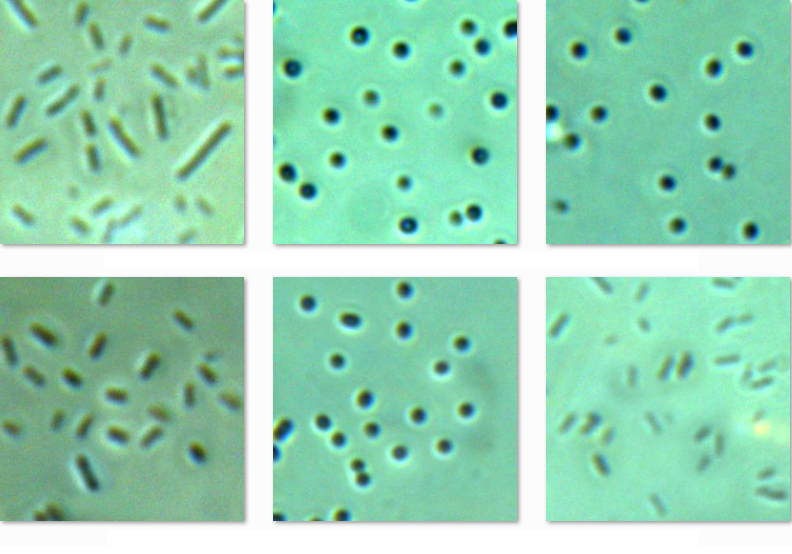


**Figure 4.2:** Colony characteristics and microscopic morphology of the isolated bacteria: **(A)** Colony characteristics on nutrient agar plate; **(B)** 100× magnified microscopic morphology

**4.1.4 Screening of the Dye Degrading Bacteria**

The screening results of the isolated bacteria are shown in Figure 4.3A–B and Table 4.3. After treatment in enrichment medium, each individual bacterium was treated in MS medium containing crystal violet 50 mg/l and mixed (crystal violet and malachite green) 15 + 25 mg/l to observe if they were able to utilize or not the specific dye in the absence of nutrients. The bacterium showed dye degrading activity was selected for screening. Treatment in MS medium containing dye, all isolates demonstrated significant dye degrading activity as shown in Figure 4.3A.

To find out the best decolorizer, the isolates according to their isolation and selection were incubated with 10% (v/v) inoculum at 35°C under shaking condition at 120 rpm in MS medium separately containing crystal violet 100 mg/l and mixed (crystal violet and malachite green) 50+50 mg/l. Figure 4.3B shows that CV–S1, CV–S2 and CV–S3 were able to degrade crystal violet and, CM–S1, CM–S2 and CM–S3 to were able to degrade mixed (crystal violet and malachite green) dye with different capacity. Table 4.3 indicates that two potential isolates namely; CV–S1 and CM–S1 showed the best degradation efficiency in crystal violet and mixed (crystal violet and malachite green) dye respectively.

The isolation of different microorganisms from the sample indicates the natural adaptation of microorganisms to survive in the presence of toxic dyes. The difference in their rate of degradation may be due to the loss of ecological interaction, which they might be sharing with each other under natural conditions (Sharma *et al.*, 2004).


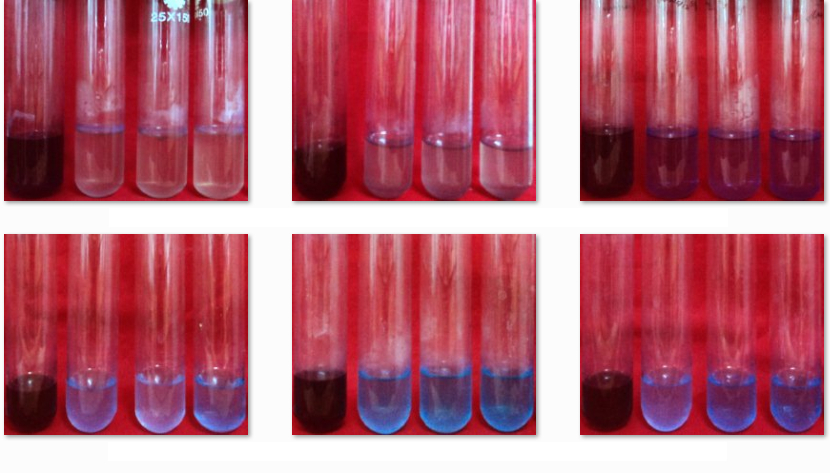


**Control**

**Control**

**Control**

**Control**

**Control**

**Control**

**A**

**B**

**C**

**F**

**E**

**D**

**Figure 4.3A:** Final selection of dye degrading isolates inoculating in MS medium after culturing in MS agar medium and sugar containing MS medium sequentially: (**A)** CV–S1, (**B)** CV–S2, (**C)** CV–S3 in MS medium responsible for crystal violet dye degradation; (**D)** CM–S1, (**E)** CM–S2, (**F)** CM–S3 in MS medium responsible for mixed (crystal violet and malachite green) dye degradation


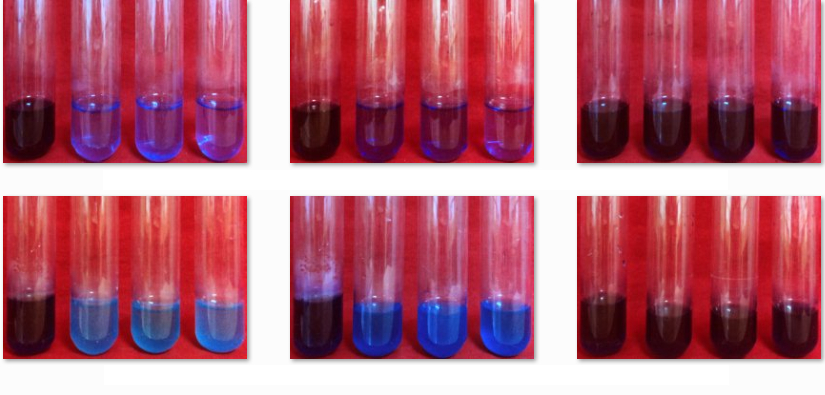


**Control**

**Control**

**Control**

**Control**

**Control**

**Control**

**A**

**B**

**C**

**F**

**E**

**D**

**Figure 4.3B:** Screening result of dye degrading bacteria in MS medium: (**A)** CV–S1, (**B)** CV–S2, (**C)** CV–S3 in MS medium responsible for crystal violet dye degradation; (**D)** CM–S1, (**E)** CM–S2, (**F)** CM–S3 in MS medium responsible for mixed (crystal violet and malachite green) dye degradation

**Table 4.3:** Screening result of dye decolorizing isolates

| **Isolates** | **Initial OD** | **Final OD** | **Degradation rate (%)** | **Average degradation rate (%)** | **Duration of observation** |
| --- | --- | --- | --- | --- | --- |
|  | 0.08 | 0.015 | 81.25 |  |  |
| CV–S1 | 0.08 | 0.015 | 81.25 | 81.25 | 72 hours |
|  | 0.08 | 0.015 | 81.25 |  |  |
|  | 0.08 | 0.03 | 62.50 |  |  |
| CV–S2 | 0.08 | 0.03 | 62.50 | 64.58 | 72 hours |
|  | 0.08 | 0.025 | 68.75 |  |  |
|  | 0.08 | 0.06 | 25.00 |  |  |
| CV–S3 | 0.08 | 0.06 | 25.00 | 25.00 | 72 hours |
|  | 0.08 | 0.06 | 25.00 |  |  |
|  | 0.20 | 0.03 | 85.00 |  |  |
| CM –S1 | 0.20 | 0.03 | 85.00 | 85.00 | 96 hours |
|  | 0.20 | 0.03 | 85.00 |  |  |
|  | 0.20 | 0.05 | 75.00 |  |  |
| CM –S2 | 0.20 | 0.05 | 75.00 | 75.00 | 96 hours |
|  | 0.20 | 0.05 | 75.00 |  |  |
|  | 0.20 | 0.10 | 50.00 |  |  |
| CM –S3 | 0.20 | 0.10 | 50.00 | 50.00 | 96 hours |
|  | 0.20 | 0.10 | 50.00 |  |  |

**4.1.5 Determination of Bacterial Growth**

Population growth was studied by analyzing the growth curve of a microbial culture. Microorganisms were cultivated in liquid medium and grown in a batch culture or closed system. Growth of microorganisms reproducing can be plotted as the logarithm of the number of viable cells versus the incubation time by binary fission as shown in Figure 4.4.

Growth curve was obtained for two highly potential dye decolorizing bacterial isolates, CV–S1 and CM–S1. They showed growth variations at different temperature and pH (Figure 4.4).

**A**

**B**

**D**

**C**

**Figure 4.4:** Effect of pH and temperature on bacterial growth: Effect of pH at 35°C on **(A)** CV–S1 and **(B)** CM–S1; Effect of temperature at pH 6.50 on **(C)** CV–S1 and **(D)** CM–S1

**4.1.6 Carbohydrate Utilization Test**

8 different carbohydrates utilization by CV–S1 and CM–S1 are shown in Figure 4.5A – 4.5B. Utilization observed in nutrient broth with respective sugar, at 35°C for 72 hours. Both bacteria revealed positive result in carbohydrate utilization. The overall results of carbohydrate utilization test of the screened bacterial strains are summarized in Table 4.4.

**Table 4.4:** Carbohydrate utilization test for screened bacterial strain

| **Tests** | **Results** | |
| --- | --- | --- |
|  | **CV–S1** | **CM–S1** |
| Arabinose | Positive | Positive |
| Xylose | Positive | Positive |
| Fructose | Positive | Positive |
| Lactose | Positive | Positive |
| Galactose | Positive | Positive |
| Sucrose | Positive | Positive |
| Maltose | Positive | Positive |
| Cellulose | Positive | Positive |

**
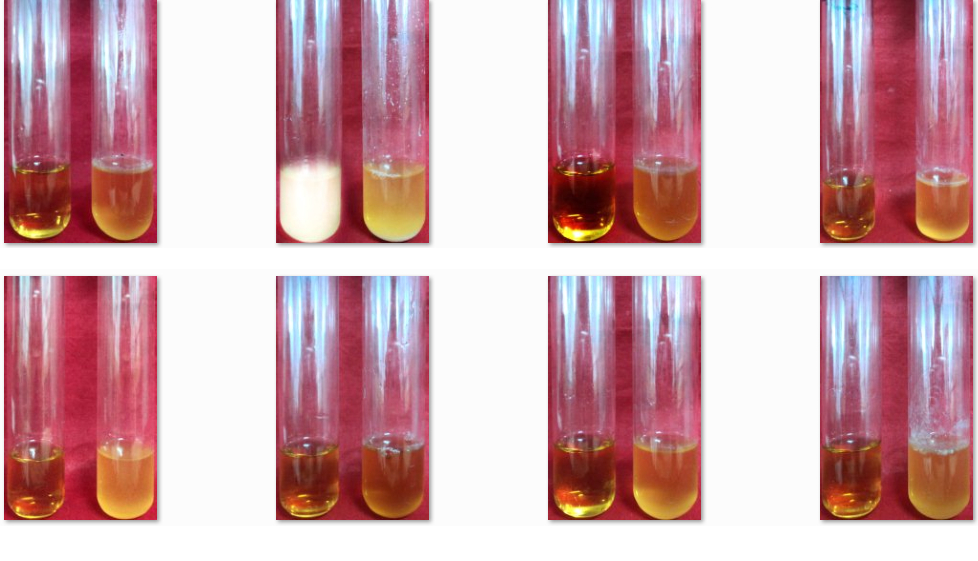
**

**Control**

**Control**

**Control**

**Control**

**Control**

**Control**

**Control**

**Control**

**A**

**H**

**G**

**F**

**E**

**B**

**C**

**D**

**Control**

**Control**

**Control**

**Control**

**Control**

**Control**

**Control**

**Control**

**A**

**H**

**G**

**F**

**E**

**B**

**C**

**D**

**Figure 4.5A:** Carbohydrate utilization test for CV–S1: (**A)** Arabinose, (**B)** Cellulose, (**C)** Fructose, (**D)** Galactose, (**E)** Lactose, (**F)** Maltose, (**G)** Sucrose, (**H)** Xylose

**
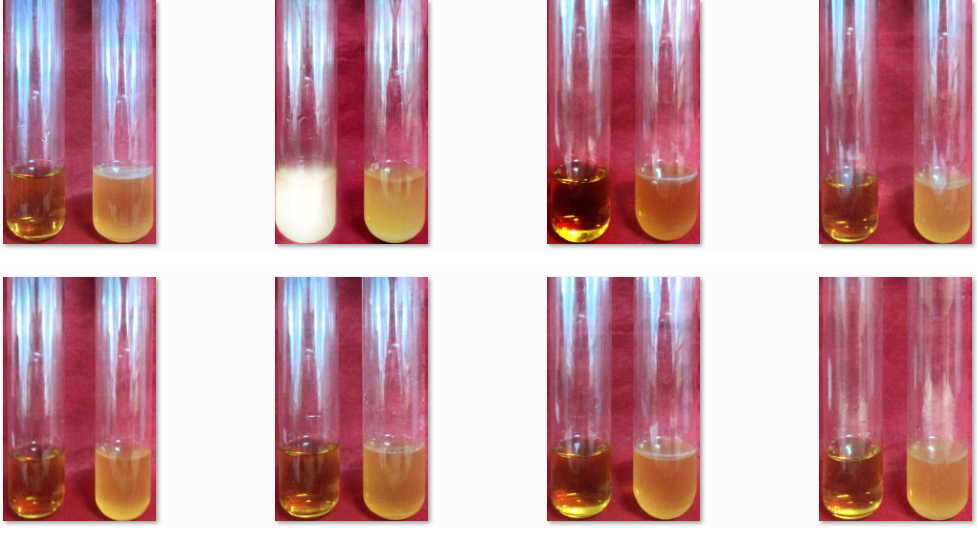
**

**Figure 4.5B:** Carbohydrate utilization test for CM–S1: (**A)** Arabinose, (**B)** Cellulose, (**C)** Fructose, (**D)** Galactose, (**E)** Lactose, (**F)** Maltose, (**G)** Sucrose, (**H)** Xylose

**4.1.7 Antibiotic Sensitivity Test**

Result of antibiotic sensitivity test on CV–S1 and CM–S1 are shown Figure 4.5 and Table 4.5. The patterns and sensitivity and resistance of isolating bacterial cultures to 10 different antibiotics were tested by disk method using nutrient agar medium. After incubation overnight at 35°C, the diameter of inhibition zone was measured.

From the Table 4.5 it is evident that the isolated bacteria are resistant to 3 antibiotics out of 10 (Figure 4.5).

**Table 4.5:** Result of antibiotic sensitivity test of CV–S1 and CM–S1

| **Antibiotics** | **CV–S1** | |  |  | **CM–S1** | |
| --- | --- | --- | --- | --- | --- | --- |
|  | **Inhibition zone (mm)** | **Sensitivity** |  |  | **Inhibition zone (mm)** | **Sensitivity** |
| Ampicilin | 11.50 | I |  |  | 13.50 | I |
| Azithromycin | 19.00 | S |  |  | 18.00 | S |
| Bacitracin | 0.00 | R |  |  | 0.00 | R |
| Cephradine | 0.00 | R |  |  | 0.00 | R |
| Cefriaxone | 18.50 | S |  |  | 18.00 | S |
| Doxycycline | 12.50 | I |  |  | 14.00 | I |
| Erythromycin | 0.00 | R |  |  | 0.00 | R |
| Neomycin | 12.00 | I |  |  | 10.50 | I |
| Sulphamethoxazole/Trimethoprim | 15.50 | S |  |  | 17.50 | S |
| Tetracycline | 17.50 | S |  |  | 16.00 | S |
| **Note:** 5–10mm = Resistant (R), 10–15mm = Intermediate resistant (I) and 15–20mm = Sensitive (S) to antibiotic | | | | | | |


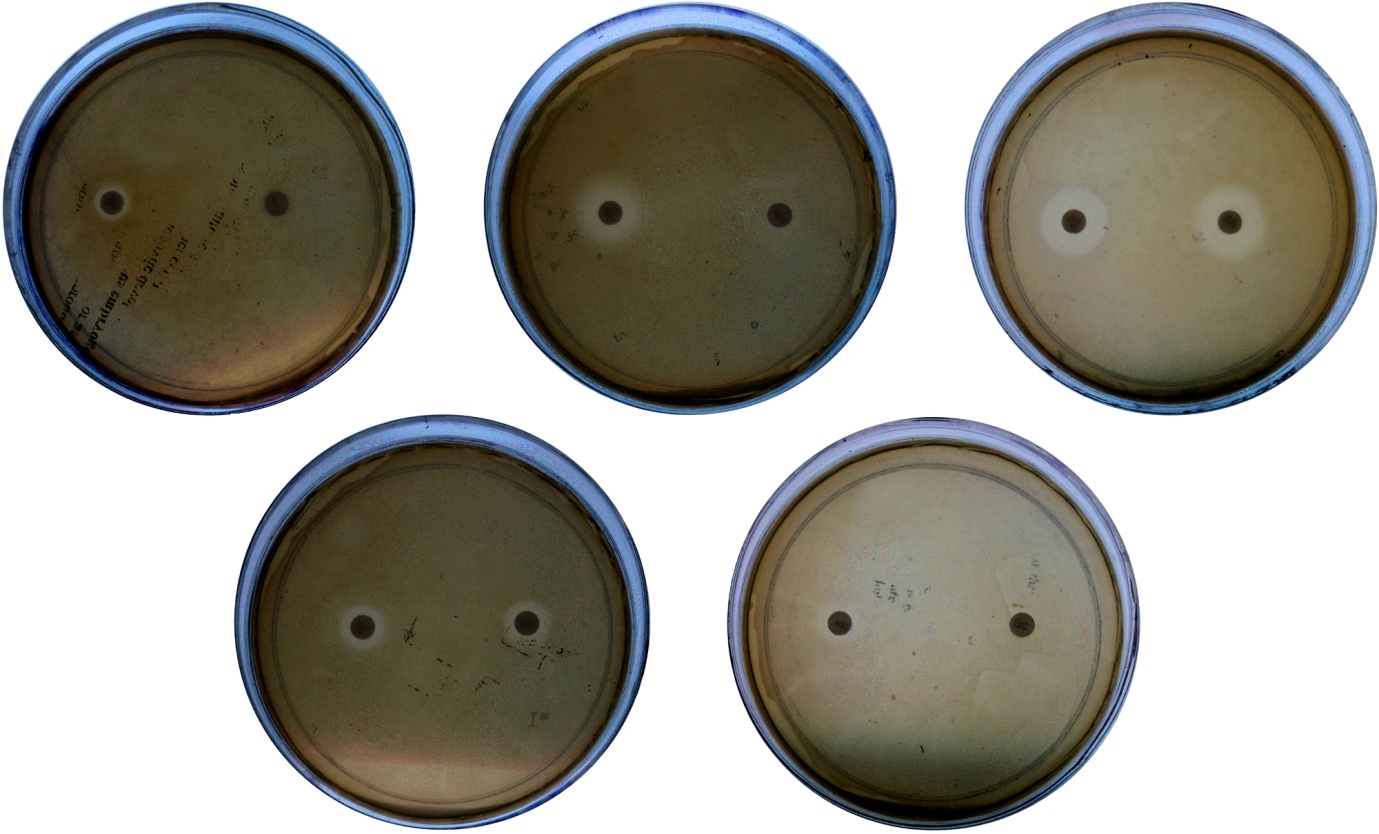


**AMP**

**CRO**

**E**

**AZM**

**TE**

**DO**

**SXT**

**N**

**CE**

**B**

**A**

**B**

**AMP**

**DO**

**E**

**AZM**

**SXT**

**N**

**CE**

**TE**

**CRO**

**B**


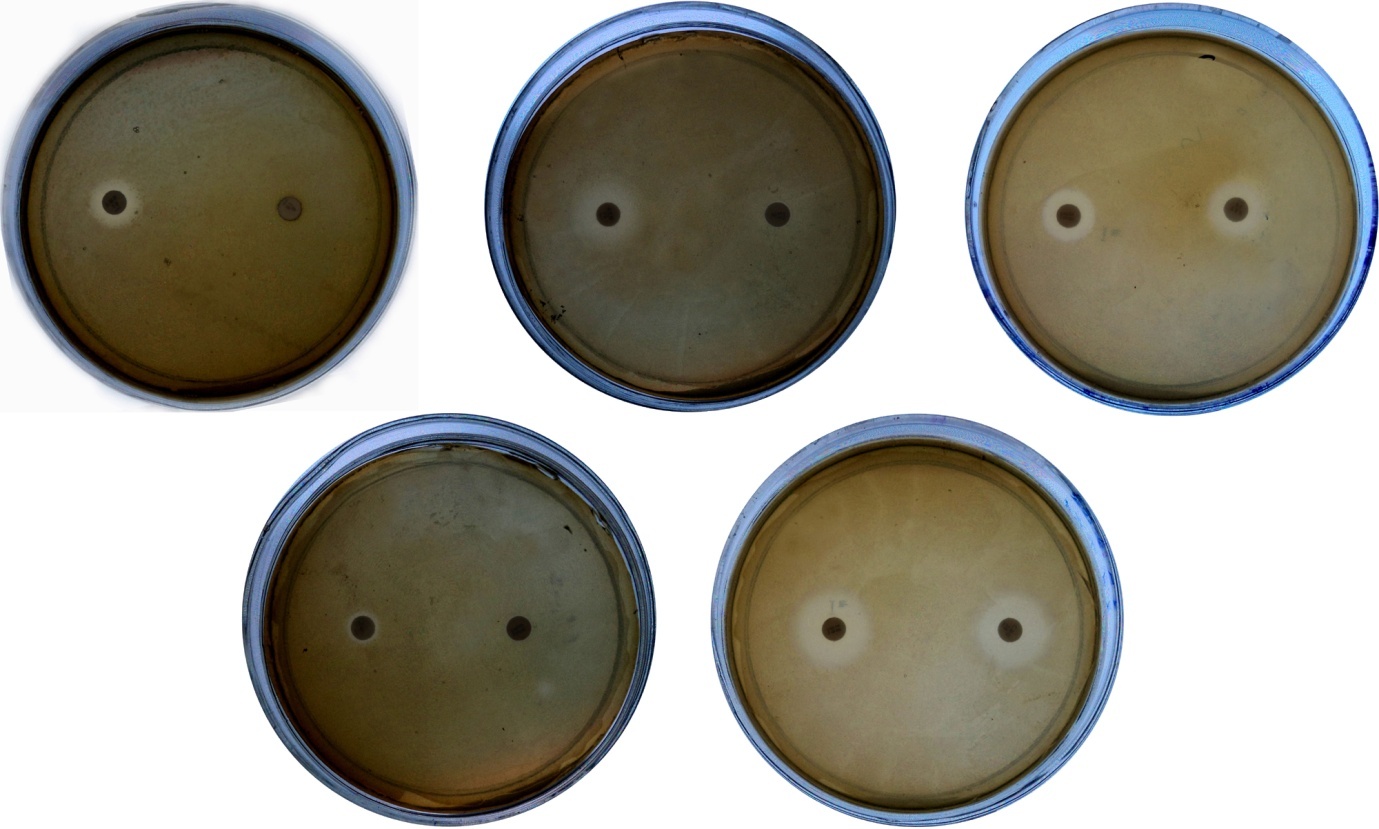


**Figure 4.5:** Antibiotic sensitivity test on nutrient agar plate: **(A)** CV–S1; **(B)** CM–S1; AMP – Ampicilin, AZM – Azithromycin, B – Bacitracin, CE – Cephradine, CRO – Cefriaxone, DO – Doxycycline, E – Erythromycin, N – Neomycin, SXT – Sulphamethoxazole/Trimethoprim, TE – Tetracycline

**4.1.8 Amplification of 16S rDNA Extracted from Isolated Bacterial Strains**

Electrophoretic analysis of the isolated DNA from two bacterial strains, CV–S1 and CMS–1, using 1% agarose gel followed by observation on Ultraviolet transilluminator revealed sharp high molecular weight bands of DNA that indicates the DNA was of good quality and suitable for PCR analysis. The 16S rDNA of the isolated bacterial strains was then amplified using bacteria specific universal primers 27F and 1391R.

Electrophoretic analysis of amplified 16S rDNA using 1.4% agarose gel followed by observation on gel documentation system (Alpha Innotech) indicates that the 16S rDNA of both bacterial strains was amplified up to 1500 bp which was confirmed by 2 kb DNA ladder (Invitrogen) as shown in Figure 4.6.

**4.1.9 RAPD Analysis of Extracted DNA from Isolated Bacterial Strains**

The variable genes of the isolated bacterial strains were amplified using three RAPD primers: MT370563, MT370571 and MT370573. Electrophoretic analysis of amplified variable genes using 1.4% agarose gel followed by observation on gel documentation system (Alpha Innotech) indicates the two isolated bacterial strains, CV–S1 and CMS–1, are genotypically different. Among three RAPD primers MT370573 primer showed more variation than MT370563 and MT370571 as shown in Figure 4.7.


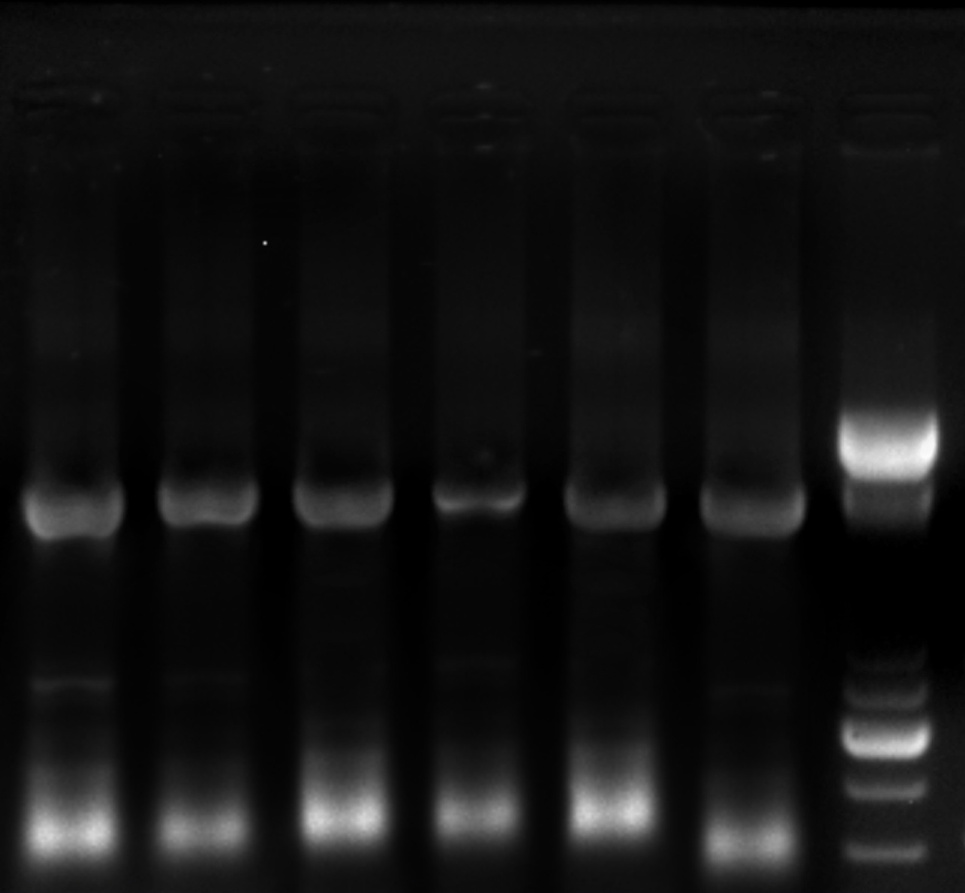


**MT370563**

**MT370571**

**MT370573**

**Ladder**

**CM–S1**

**CV–S1**

**CM–S1**

**CV–S1**

**CV–S1**

**CM–S1**

**600 bp**

**1500 bp**

**2072 bp**

**CM–S1**

**CM–S1**

**CM–S1**

**CV–S1**

**CV–S1**

**Ladder**

**CV–S1**

**600 bp**

**1500 bp**

**2072 bp**

**Figure 4.6:** Amplified PCR products of 16S rDNA (1500bp).

**
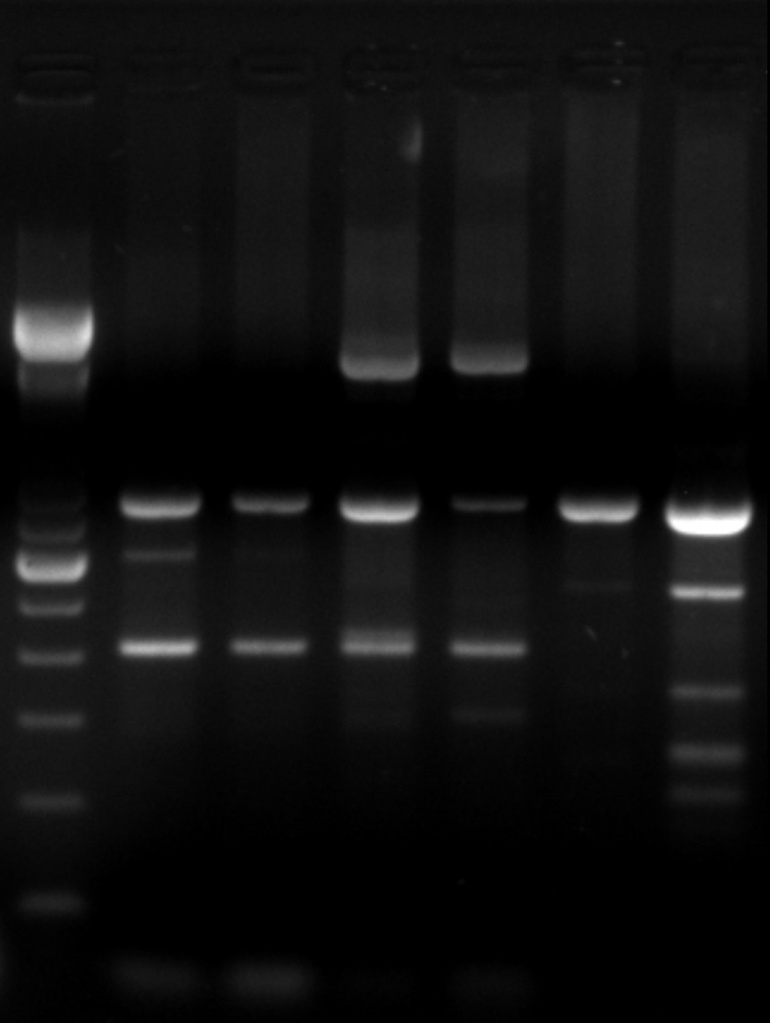
**

**Figure 4.7:** Observation of genotypic variation of two isolates using three RAPD primers

**4.1.10 16S rDNA Sequencing Analysis and Phylogenic Tree**

The use of 16S rDNA sequences to study bacterial phylogeny and taxonomy has been by far the most common housekeeping genetic marker used for a number of reasons. These reasons include (i) its presence in almost all bacteria, often existing as a multigene family, or operons; (ii) the function of the 16S rDNA over time has not changed, suggesting that random sequence changes are a more accurate measure of time (evolution); and (iii) the 16S rRNA gene (1,500 bp) is large enough for informatics purposes **(Patel, 2001**).

16S rDNA sequences as shown in Table 4.6a were determined for two different bacterial isolates namely, CV–S1 and CM–S1. These sequences were submitted to NCBI. Highest identity 99% according to isolation source for CV–S1 was *Enterobacter* sp. HSL69 and for CM–S1 was *Enterobacter* sp. HSL99. To analyze the phylogenetic position, the 16S rDNA sequence of the strain CV–S1 (580 bp) and CM–S1 (580 bp) were determined. Figure 4.8 showed the phylogenetic relationship between the isolated bacterial strains and other related bacteria, shown in Table 4.6b with their similarity, found in the GenBank database. The homology indicated that the strain CM–S1 was in the phylogenetic branch of the genus *Enterobacter*, and the strain CV–S1 formed a new branch. These isolates were identified as *Enterobacter* sp. CV–S1 and *Enterobacter* sp. CM–S1. The newly formed branch confirms that the identified *Enterobacter* sp. CV–S1 is new species of *Enterobacter* genus.

**Table 4.6A:** 16S rDNA sequences of the isolated bacterial strains

| **CV–S1** | **CM–S1** |
| --- | --- |
| CGTGCATTCTGATCTACGATTACTAGCGATTCCGACTTCATGGAGTCGAGTTGCAGACTCCAATCCGGACTACGACGCACTTTATGAGGTCCGCTTGCTCTCGCGAGGTCGCTTCTCTTTGTATGCGCCATTGTAGCACGTGTGTAGCCCTGGTCGTAAGGGCCATGATGACTTGACGTCATCCCCACCTTCCTCCAGTTTATCACTGGCAGTCTCCTTTGAGTTCCCGGCCTGACCGCTGGCAACAAAGGATAAGGGTTGCGCTCGTTGCGGGACTTAACCCAACATTTCACAACACGAGCTGACGACAGCCATGCAGCACCTGTCTCACAGTTCCCGAAGGCACCAAAGCATCTCTGCTAAGTTCTGTGGATGTCAAGACCAGGTAAGGTTCTTCGCGTTGCATCGAATTAAACCACATGCTCCACCGCTTGTGCGGGCCCCCGTCAATTCATTTGAGTTTTAACCTTGCGGCCGTACTCCCCAGGCGGTCGATTTAACGCGTTAGCTCCGGAAGCCACGCCCTCAAGGGCACAACCTCCCAAATCGACATCGTTTACGGCGTGGAACTACCAAGGTA | GCAGTCGACGGTAGCACAGAGAGCTTGCTCTCGGGTGACGAGTGGCGGACGGGTGAGTAATGTCTGGGAAACTGCCTGATGGAGGGGGATAACTACTGGAAACGGTAGCTAATACCGCATAACGTCGCAAGACCAAAGAGGGGGACCTTCGGGCCTCTTGCCATCAGATGTGCCCAGATGGGATTAGCTAGTAGGTGGGGTAACGGCTCACCTAGGCGACGATCCCTAGCTGGTCTGAGAGGATGACCAGCCACACTGGAACTGAGACACGGTCCAGACTCCTACGGGAGGCAGCAGTGGGGAATATTGCACAATGGGCGCAAGCCTGATGCAGCCATGCCGCGTGTATGAAGAAGGCCTTCGGGTTGTAAAGTACTTTCAGCGGGGAGGAAGGTGTTGTGGTTAATAACCGCAGCAATTGACGTTACCCGCAGAAGAAGCACCGGCTAACTCCGTGCCAGCAGCCGCGGTAATACGGAGGGTGCAAGCGTTAATCGGAAATTACTGGGCGTAAAGCGCACGCAGGCGGTCTGTCAAGTCGGATGTGAAATCCCCGGGCTCAACCTGGGAACTGCATTCG |

**Table 4.6B:** Similarity between the isolated bacterial strains CV–S1 and CM–S1; and other related bacteria found in the GenBank database

| Isolated strain | Closed bacteria | Accession no. | Identity (%) |
| --- | --- | --- | --- |
| CV–S1 | *Enterobacter cloacae* RU14 | KJ607595.1 | 99 |
|  | *Enterobacter cloacae* RJ04 | KC990807.1 | 99 |
|  | *Enterobacter* sp. HSL69 | HM461195.1 | 99 |
|  | *Enterobacter* *sacchari* SP1 | NR_118333.1 | 98 |
| CM–S1 | *Enterobacter cloacae* TPL2 | KJ470636.1 | 99 |
|  | *Enterobacter* sp. HSL99 | HM461229.1 | 99 |
|  | *Enterobacter* sp. HSL76B | HM461202.1 | 99 |
|  | *Enterobacter* *cloacae* RM20 | KJ607605.1 | 99 |


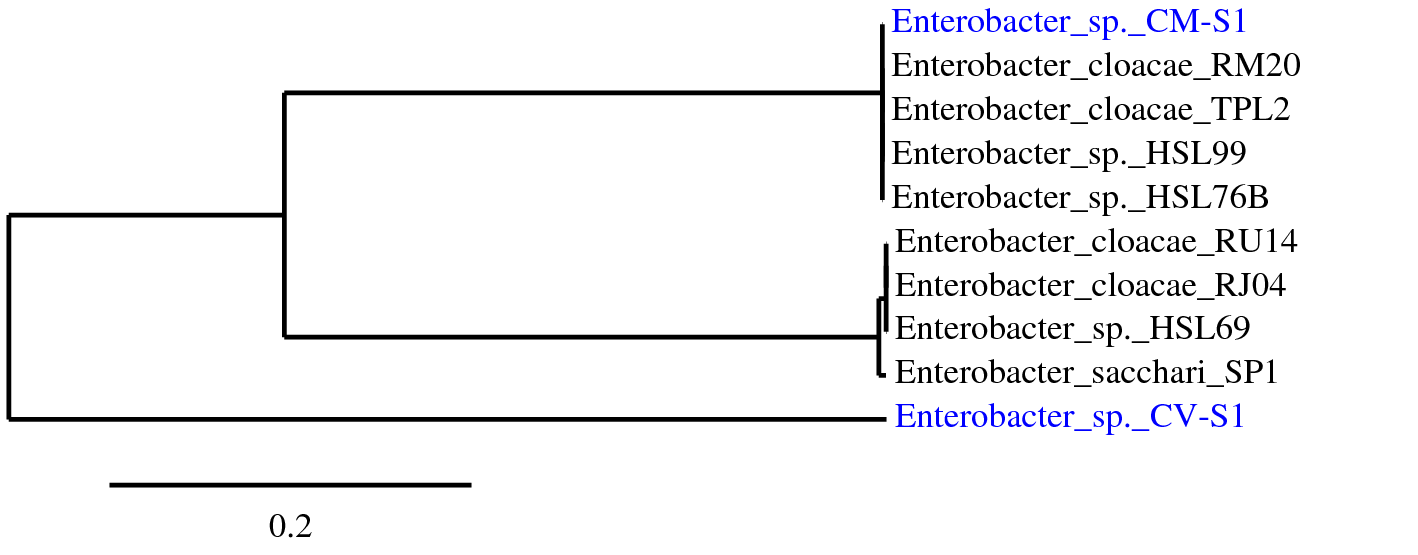


**Figure 4.8:** Phylogenetic tree analysis: The evolutionary history was inferred using the Neighbor–Joining method. Highlighted bacterial strains are the isolated bacteria. The phylogenetic tree was reconstructed using the maximum likelihood method implemented in the PhyML program (v3.0 aLRT) ((Dereeper *et al.*, 2010; Edgar, 2004).

**4.2 Influence of Environmental Parameters on the Process of Textile Dye Degradation**

Textile effluent is rich source of dye decolorizing bacterial population. Total 6 isolates were selected on the basis of their colony characteristics. Initially, all 6 isolates were tested for their ability to degrade two triphenylmethane dyes, crystal violet and mixed (crystal violet and malachite green). Finally, 2 potential isolates were selected on the basis of their dye decolorizing ability and identified as *Enterobacter* sp. CV–S1 for crystal violet and *Enterobacter* sp. CM–S1 for mixed (crystal violet and malachite green) dye degradation.

Thus, in this section, the results of degradation experiment of crystal violet dye by *Enterobacter* sp. CV–S1 and mixed (crystal violet and malachite green) dye by *Enterobacter* sp. CM–S1 was organized considering the effect of pH, temperature, initial dye concentration and inoculum size under aerobic shaking condition at 120rpm.

**4.2.1 Effect of pH on Dye Degradation**

The pH tolerance is an important consideration for industrial applications. At lower pH values, the H^+^ ions compete effectively with dye cations, causing a decrease in color removal efficiency. Furthermore, at high pH, the surface of biomass gets negatively charged, which enhance the positively charged dye cations through electrostatic force of attraction (Lamia *et al*., 2009).

The experiment was performed in 50 ml test tubes containing 10 ml MS medium containing 50 mg/l violet dye and 25 + 12.5 mg/l mixed (crystal violet and malachite green) dye. Due to lower degradation rate of malachite green than crystal violet as shown in Figure 4.3B, 2:1 ratio of crystal violet and malachite green was used for mixed dye treatment. The findings of the effect of pH on dye degradation are presented in Table 4.7A–B Figure 4.9A–B and 4.10A–B.

It was observed that the percentage of crystal violet dye degradation varied with change in pH of the medium (Figure 4.9A). Prominent degradation was occurred around pH 6.50 to 7.00 and highest decolorization rate 100% was observed highest at pH 6.50 and lowest 12.5% at pH 7.00 (Figure 4.9A–B, Table 4.7A). However, organism showed very poor decolorization at above pH 7.50 – 8.50.

On the other hand, at lower concentration the mixed dye degradation was occurred in a wide range of pH (Figure 4.10A). After 24 hours approximately similar transparencies were observed from pH 6.50–8.50 except pH 6.00 but higher rate of degradation 100% was occurred at pH 6.50 and with the increasing of pH the rate of degradation decreased (Table 4.7B and Figure 4.10A–B). There is also a matter of fact that with the increasing of pH the mixed dye solution undergoes considerably gradual auto–degradation (Table 4.7B). But in case of crystal violet, it was observed that crystal violet can withstand its color in a wide range of pH (Table 4.7A). So, it is clear that the significant auto–degradation at higher pH was due to acidic nature of malachite green which ultimately established an acid–base neutral reaction; thus at higher pH the initial absorbance were decreased gradually. There is also a considerable matter that at higher pH *Enterobacter* sp. CM–S1 had little contribution on dye degradation due to occurring chemical degradation.

These observations indicate that the organism can treat weakly acidic to neutral dyeing waste water at normal operational pH and decrease the cost of acidification or basification.

**Table 4.7A:** Effect of pH on crystal violet dye decolorization by *Enterobacter* sp. CV–S1

| **pH** | **Initial OD** | **Final OD** | **Degradation rate (%)** | **Average Degradation rate (%)** | **Duration of observation** |
| --- | --- | --- | --- | --- | --- |
|  | 0.04 | 0.035 | 12.5 |  |  |
| 6.00 | 0.04 | 0.035 | 12.5 | 12.5 | 24 hours |
|  | 0.04 | 0.035 | 12.5 |  |  |
|  | 0.04 | 0.00 | 100 |  |  |
| 6.50 | 0.04 | 0.00 | 100 | 100 | 24 hours |
|  | 0.04 | 0.00 | 100 |  |  |
|  | 0.04 | 0.005 | 87.5 |  |  |
| 7.00 | 0.04 | 0.005 | 87.5 | 87.5 | 24 hours |
|  | 0.04 | 0.005 | 87.5 |  |  |
|  | 0.04 | 0.025 | 37.5 |  |  |
| 7.50 | 0.04 | 0.025 | 37.5 | 37.5 | 24 hours |
|  | 0.04 | 0.025 | 37.5 |  |  |
|  | 0.04 | 0.03 | 25.00 |  |  |
| 8.00 | 0.04 | 0.03 | 25.00 | 25.00 | 24 hours |
|  | 0.04 | 0.03 | 25.00 |  |  |
|  | 0.04 | 0.03 | 25.00 |  |  |
| 8.50 | 0.04 | 0.03 | 25.00 | 25.00 | 24 hours |
|  | 0.04 | 0.03 | 25.00 |  |  |

**Table 4.7B:** Effect of pH on mixed (crystal violet and malachite green) dye degradation by *Enterobacter* sp. CM–S1

| **pH** | **Initial OD** | **Final OD** | **Degradation rate (%)** | **Average Degradation rate (%)** | **Duration of observation** |
| --- | --- | --- | --- | --- | --- |
|  | 0.19 | 0.11 | 42.10 |  |  |
| 6.00 | 0.19 | 0.12 | 36.84 | 47.37 | 24 hours |
|  | 0.19 | 0.07 | 63.16 |  |  |
|  | 0.19 | 0.00 | 100 |  |  |
| 6.50 | 0.19 | 0.00 | 100 | 100 | 24 hours |
|  | 0.19 | 0.00 | 100 |  |  |
|  | 0.14 | 0.002 | 98.57 |  |  |
| 7.00 | 0.14 | 0.002 | 98.57 | 98.57 | 24 hours |
|  | 0.14 | 0.002 | 98.57 |  |  |
|  | 0.10 | 0.002 | 98.00 |  |  |
| 7.50 | 0.10 | 0.002 | 98.00 | 98.00 | 24 hours |
|  | 0.10 | 0.002 | 98.00 |  |  |
|  | 0.08 | 0.005 | 93.75 |  |  |
| 8.00 | 0.08 | 0.005 | 93.75 | 93.75 | 24 hours |
|  | 0.08 | 0.005 | 93.75 |  |  |
|  | 0.08 | 0.005 | 93.75 |  |  |
| 8.50 | 0.08 | 0.005 | 93.75 | 93.75 | 24 hours |
|  | 0.08 | 0.005 | 93.75 |  |  |

**
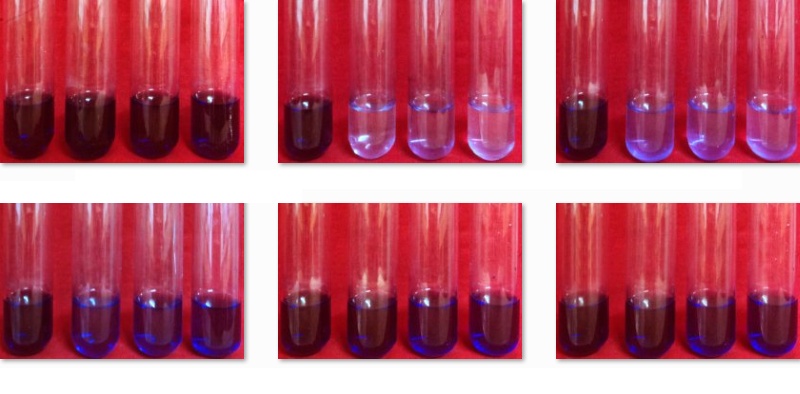
**

**Control**

**Control**

**Control**

**Control**

**Control**

**Control**

**A**

**D**

**E**

**F**

**C**

**B**

**Figure 4.9A:** Effect of pH on crystal violet dye degradation by *Enterobacter* sp. CV–S1: (**A)** pH 6.00, (**B)** pH 6.50, (**C)** pH 7.00, (**D)** pH 7.50, (**E)** pH 8.00, (**F)** pH 8.50

**Figure 4.9B:** Graphical presentation of the effect of pH on crystal violet dye degradation by *Enterobacter* sp. CV–S1

**
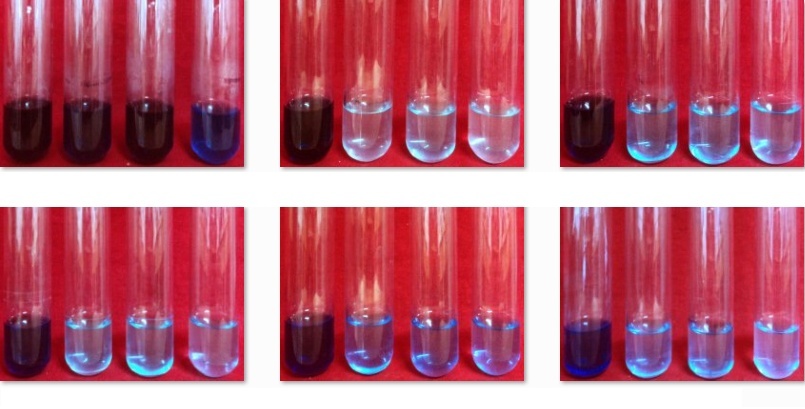
**

**Control**

**Control**

**Control**

**Control**

**Control**

**Control**

**A**

**D**

**E**

**F**

**C**

**B**

**Figure 4.10A:** Effect of pH on mixed (crystal violet and malachite green) dye degradation by *Enterobacter* sp. CM–S1: (**A)** pH 6.00, (**B)** pH 6.50, (**C)** pH 7.00, (**D)** pH 7.50, (**E)** pH 8.00, (**F)** pH8.50

**Figure 4.10B:** Graphical presentation of the effect of pH on mixed (crystal violet and malachite green) dye degradation by *Enterobacter* sp. CM–S1

**4.2.2 Effect of Temperature on Dye Degradation**

The mesophilic range is traditionally used since it is generally thought that maintaining high temperature would be uneconomical, while dye degradation within the psychrophilic range is too slow (Varel *et al*., 1980).

In order to determine the optimum temperature, degradation assay were performed around 30 – 40°C temperature range at pH 6.50 using 50 mg/l crystal violet and 25 + 12.5 mg/l mixed (crystal violet and malachite green) dye respectively. The results of the effect of temperature on dye degradation are presented in Table 4.8A–B, Figure 4.11A–B and 4.12A–B. After 24 hours it was observed that in both cases the maximum degradation rate 100% had occurred at temperature 35°C (Figure 4.11A and Figure 4.12A). At 30°C and 40°C there was much adverse effect on the degradation (Figure 4.11B and Figure 4.12B). This might have occurred due to adverse effect of lower and higher temperature other than 35°C on the enzymatic activities.

**Table 4.8A:** Effect of temperature on crystal violet dye degradation by *Enterobacter* sp. CV–S1

| **Temperature** | **Initial OD** | **Final OD** | **Degradation rate (%)** | **Average degradation rate (%)** | **Duration of observation** |
| --- | --- | --- | --- | --- | --- |
|  | 0.04 | 0.025 | 37.5 |  |  |
| 30°C | 0.04 | 0.025 | 37.5 | 37.5 | 24 hours |
|  | 0.04 | 0.025 | 37.5 |  |  |
|  | 0.04 | 0.00 | 100 |  |  |
| 35°C | 0.04 | 0.00 | 100 | 100 | 24 hours |
|  | 0.04 | 0.00 | 100 |  |  |
|  | 0.04 | 0.025 | 37.5 |  |  |
| 40°C | 0.04 | 0.025 | 37.5 | 37.5 | 24 hours |
|  | 0.04 | 0.025 | 37.5 |  |  |

**Table 4.8B:** Effect of temperature on mixed (crystal violet and malachite green) dye degradation by *Enterobacter* sp. CM–S1

| **Temperature** | **Initial OD** | **Final OD** | **Degradation rate (%)** | **Average degradation rate (%)** | **Duration of observation** |
| --- | --- | --- | --- | --- | --- |
|  | 0.19 | 0.08 | 57.89 |  |  |
| 30°C | 0.19 | 0.08 | 57.89 | 57.89 | 24 hours |
|  | 0.19 | 0.08 | 57.89 |  |  |
|  | 0.19 | 0.00 | 100 |  |  |
| 35°C | 0.19 | 0.00 | 100 | 100 | 24 hours |
|  | 0.19 | 0.00 | 100 |  |  |
|  | 0.19 | 0.09 | 52.63 |  |  |
| 40°C | 0.19 | 0.09 | 52.63 | 52.63 | 24 hours |
|  | 0.19 | 0.09 | 52.63 |  |  |


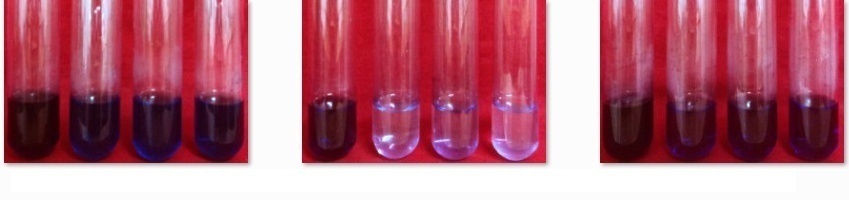


**Control**

**Control**

**Control**

**A**

**C**

**B**

**Figure 4.11A:** Effect of temperature on crystal violet dye degradation by *Enterobacter* sp. CV–S1 (after 24 hours). (**A)** Temperature 30°C, (**B)** Temperature 35°C, (**C)** Temperature 40°C.

**Figure 4.11B:** Graphical presentation of the effect of temperature on crystal violet dye degradation by *Enterobacter* sp. CV–S1


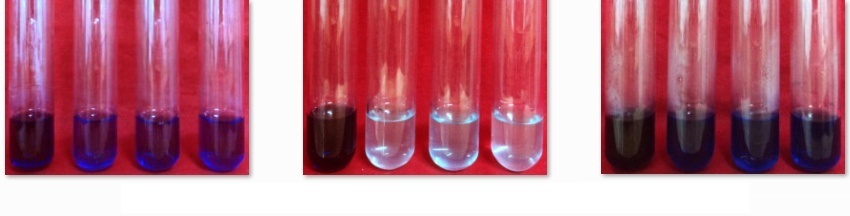


**Control**

**Control**

**Control**

**A**

**C**

**B**

**Figure 4.12A:** Effect of temperature on mixed (crystal violet and malachite green) dye degradation by *Enterobacter* sp. CM–S1 (after 24 hours). (**A)** Temperature 30°C, (**B)** Temperature 35°C, (**C)** Temperature 40°C.

**Figure 4.12B:** Graphical presentation of the effect of temperature on mixed (crystal violet and malachite green) dye degradation by *Enterobacter* sp. CM–S1.

**4.2.3 Effect of Initial Dye Concentration on Dye Degradation**

In order to study the effect of initial concentration of crystal violet and mixed (crystal violet and malachite green) dye, the experiments were carried out at a fixed biomass (10% inoculums v/v) at different dye concentrations (50, 100, 150 and 200 mg/l of crystal violet and 25 + 12.5, 50 + 25, 75 + 37.5 mg/l of mixed (crystal violet and malachite green) for different time intervals (24, 48 and 72 hrs) at 35°C under shaking culture condition. Detail findings of the effect of temperature on dye degradation are presented in Table 4.9A–B and Figure 4.13A-B and Figure 4.14A-B.

The degradation of crystal violet at the highest (200 mg/l) and medium to lower dye concentration (50, 100 and 150 mg/l) was 43.75% and 100%, respectively (Table 4.9A and Figure 4.13A). In case of *Enterobacter* sp. CM–S1, it was able to degrade completely up to 50 + 25 mg/l within 72 hours (Table 4.9B and Figure 4.14A).

**Table 4.9A:** Effect of initial crystal violet dye concentration on degradation by *Enterobacter* sp. CV–S1

| **Dye concentration ( mg/l)** | **Initial OD** | **Final OD** | **Degradation rate (%)** | **Average Degradation rate (%)** | **Duration of observation** |
| --- | --- | --- | --- | --- | --- |
|  | 0.04 | 0.00 | 100 |  |  |
| 50 | 0.04 | 0.00 | 100 | 100 | 24 hours |
|  | 0.04 | 0.00 | 100 |  |  |
|  | 0.08 | 0.00 | 100 |  |  |
| 100 | 0.08 | 0.00 | 100 | 100 | 48 hours |
|  | 0.08 | 0.00 | 100 |  |  |
|  | 0.12 | 0.00 | 100 |  |  |
| 150 | 0.12 | 0.00 | 100 | 100 | 72 hours |
|  | 0.12 | 0.00 | 100 |  |  |
|  | 0.16 | 0.09 | 43.75 |  |  |
| 200 | 0.16 | 0.09 | 43.75 | 43.75 | 72 hours |
|  | 0.16 | 0.09 | 43.75 |  |  |

**Table 4.9B:** Effect of initial mixed (crystal violet and malachite green) dye concentration on degradation by *Enterobacter* sp. CM–S1

| **Dye concentration [(*CV + **MG) mg/l]** | **Initial OD** | **Final OD** | **Degradation rate (%)** | **Average Degradation rate (%)** | **Duration of observation** |
| --- | --- | --- | --- | --- | --- |
|  | 0.19 | 0.00 | 100 |  |  |
| 25 + 12.5 | 0.19 | 0.00 | 100 | 100 | 24 hours |
|  | 0.19 | 0.00 | 100 |  |  |
|  | 0.22 | 0.00 | 100 |  |  |
| 50 + 25 | 0.22 | 0.00 | 100 | 100 | 72 hours |
|  | 0.22 | 0.00 | 100 |  |  |
|  | 0.24 | 0.09 | 62.5 |  |  |
| 75 + 37.5 | 0.24 | 0.09 | 62.5 | 63.89 | 72 hours |
|  | 0.24 | 0.08 | 66.67 |  |  |
| *CV – Crystal violet  **MG – Malachite green | |  |  |  |  |

In both cases at lower concentrations dye degradation required relatively short time than higher. More than the concentration 150 mg/l of crystal violet and 50 + 25 mg/l of mixed (crystal violet and malachite green), the rate of degradation was decreased (Figure 4.13B and Figure 4.14B).

The decreasing rate of crystal violet and malachite green dye degradation at higher concentration due to decreasing the nucleic acids content ratio, *i.e.*, RNA/DNA, resulting lowering the protein synthesis than inhibit cell division which support the findings, reported by Ogawa *et al*. (1988), so the effect of dye concentration on growth of organisms is an important consideration for its field application. Khehra *et al.*, (2005) also suggested that the decrease in degradation efficiency might be due to the toxic effect of dyes. Furthermore, Initial concentration provides an important driving force to overcome all mass transfer resistance of the dye between the aqueous and solid phases (Parshetti *et al*., 2006).


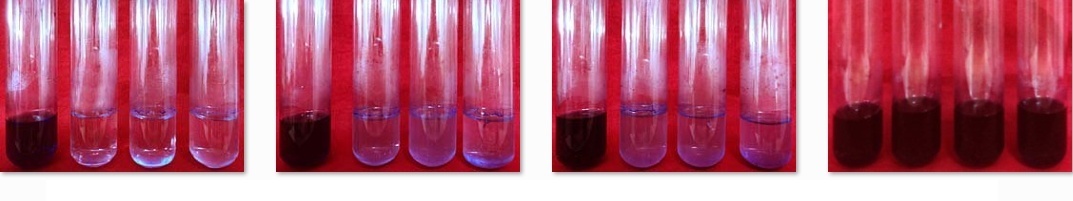


**Control**

**Control**

**Control**

**Control**

**A**

**B**

**C**

**D**

**Figure 4.13A:** Effect of initial crystal violet dye concentration on degradation by *Enterobacter* sp. CV–S1: (**A**) 50 mg/l, (**B)** 100 mg/l, (**C)** 150 mg/l, (**D)** 200 mg/l

**Figure 4.13B:** Graphical presentation of the effect of initial crystal violet dye concentration on degradation by *Enterobacter* sp. CV–S1

**
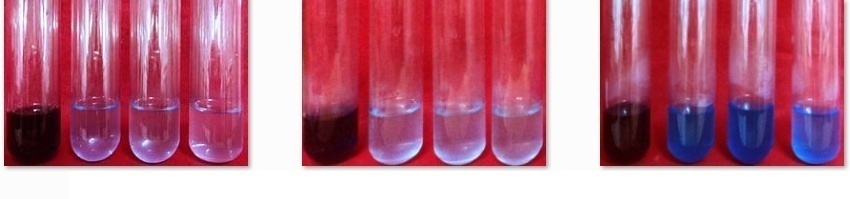
**

**Control**

**Control**

**Control**

**A**

**B**

**C**

**Figure 4.14A:** Effect of initial mixed (crystal violet and malachite green) dye concentration on degradation by *Enterobacter* sp. CM–S1: (**A)** 37.5 (25 + 12.5) mg/l, (**B)** 75 (50 + 25) mg/l, (**C)** 112.5 (75 + 37.5) mg/l

**Figure 4.14B:** Graphical presentation of the effect of initial mixed (crystal violet and malachite green) dye concentration on degradation by *Enterobacter* sp. CM–S1

**4.2.4 Effect of Initial Inoculum Size on Dye Degradation**

To estimate the optimum initial biomass of *Enterobacter* sp. CV–S1 and *Enterobacter* sp. CM–S1, for 150 mg/l of crystal violet and 50 + 25 mg/l of mixed (crystal violet and malachite green) dye concentration, different inoculum sizes containing 8, 9 and 10% (v/v) containing 27×10^9^ CFU/ml (*Enterobacter* sp. CV–S1) and 10×10^9^ CFU/ml (*Enterobacter* sp. CM–S1) were applied at pH 6.50 and 35°C. Detail results of the effect of initial inoculum size on dye degradation are shown in Table 4.10A–B and Figure 4.15A–B and 4.16A–B.

It was found that that, under shaking cultural condition, the dye removal capacity had affected by the inoculum size used (Figure 4.15A and Figure 4.16A). The degradation rate decreased with the decreasing inoculum sizes (Figure 4.15B and Figure 4.16B). Most significant result obtained when 10% inoculums was used (Table 4.10A and Table 4.10B). Similar pattern was observed by Lamia *et al.* (2009) who reported that there dye removal capacity increased significantly with the increase in inoculum size. This observation was contradictory to a report where was no proportionate increase in degradation with increase in inoculum size of *Kurthia* sp. for the treatment of the textile effluent (Sani and Banerjee, 1999).

**Table 4.10A:** Effect of inoculum size on crystal violet dye degradation by *Enterobacter* sp. CV–S1

| **Inoculum size [(%) v/v]** | **Initial OD** | **Final OD** | **Degradation rate (%)** | **Average Degradation rate (%)** | **Duration of observation** |
| --- | --- | --- | --- | --- | --- |
|  | 0.12 | 0.01 | 91.67 |  |  |
| 8 | 0.12 | 0.01 | 91.67 | 91.67 | 72 hours |
|  | 0.12 | 0.01 | 91.67 |  |  |
|  | 0.12 | 0.005 | 95.83 |  |  |
| 9 | 0.12 | 0.005 | 95.83 | 95.83 | 72 hours |
|  | 0.12 | 0.005 | 95.83 |  |  |
|  | 0.12 | 0.00 | 100 |  |  |
| 10 | 0.12 | 0.00 | 100 | 100 | 72 hours |
|  | 0.12 | 0.00 | 100 |  |  |

**Table 4.10B:** Effect of inoculum size on mixed (crystal violet and malachite green) dye degradation by *Enterobacter* sp. CM–S1

| **Inoculum size [(%) v/v]** | **Initial OD** | **Final OD** | **Degradation rate (%)** | **Average Degradation rate (%)** | **Duration of observation** |
| --- | --- | --- | --- | --- | --- |
|  | 0.22 | 0.02 | 90.91 |  |  |
| 8 | 0.22 | 0.02 | 90.91 | 90.91 | 72–96 hours |
|  | 0.22 | 0.02 | 90.91 |  |  |
|  | 0.22 | 0.01 | 95.45 |  |  |
| 9 | 0.22 | 0.01 | 95.45 | 95.45 | 72–96 hours |
|  | 0.22 | 0.01 | 95.45 |  |  |
|  | 0.22 | 0.00 | 100 |  |  |
| 10 | 0.22 | 0.00 | 100 | 100 | 72–96 hours |
|  | 0.22 | 0.00 | 100 |  |  |


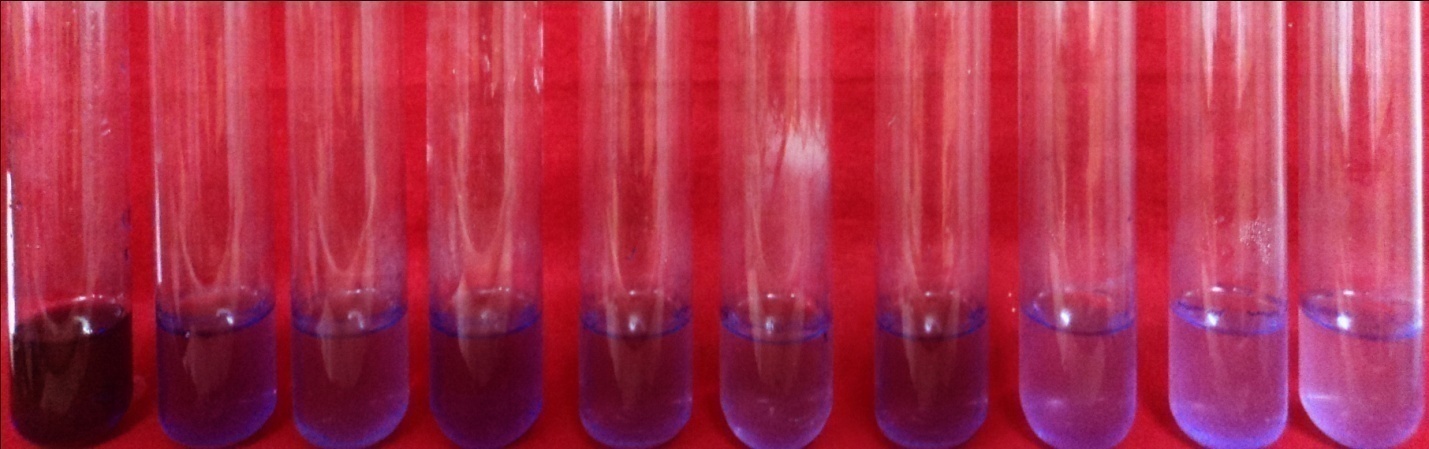


**Control**

**8%**

**9%**

**10%**

**Figure 4.15A:** Effect of inoculum size on crystal violet dye degradation by *Enterobacter* sp. CV–S1 (after 72 hours)

**Figure 4.15B:** Graphical presentation of the effect of inoculum size on crystal violet dye degradation by *Enterobacter* sp. CV–S1

**
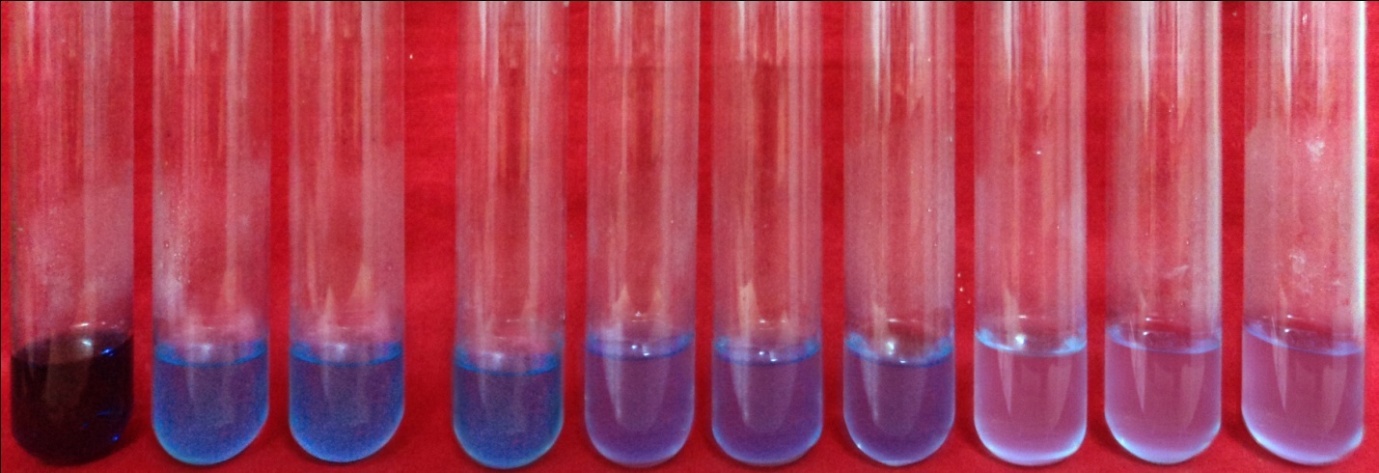
**

**Control**

**8%**

**9%**

**10%**

**Figure 4.16A:** Effect of inoculum size on mixed (crystal violet and malachite green) dye degradation by *Enterobacter* sp. CM–S1 (after 72–96 hours)

**Figure 4.16B:** Graphical presentation of the effect of inoculum size on mixed (crystal violet and malachite green) dye degradation by *Enterobacter* sp. CM–S1

**4.2.5 Malachite Green Dye Treatment by *Enterobacter* sp. CV–S1**

In the section 4.1.5 the growth characteristics of *Enterobacter* sp. CV–S1 was observed. *Enterobacter* sp. CV–S1 showed highest growth rate at pH 6.50 and 35°C. Thus in this section those growth parameters were used to observe the degradation effect of malachite green (MG) by *Enterobacter* sp. CV–S1. Results observed on the degradation of malachite green dye are presented in Table 4.11 and Figure 4.17A–C.

5% (v/v) inoculum of *Enterobacter* sp. CV–S1 showed 100% degradation of malachite green (15 mg/l) within 72 hours under shaking condition (Figure 4.17A). The degradation of malachite green was studied at various increasing concentration of dye *i.e*. from 15, 30 and 50 mg/l. It was found that the rate of degradation was decreased with increasing concentration of dye (Table 4.11 and Figure 4.17B). 77.27% and 62.5% degradation was observed at 30 and 50 mg/l dye concentration respectively. These results indicate toxicity of malachite green at higher dye concentration. At different time intervals malachite green dye degradation rate by *Enterobacter* sp. CV–S1 is shown in Figure 4.17C.

**Table 4.11:** Effect of malachite green dye concentration on degradation by *Enterobacter* sp. CV–S1

| **Dye concentration ( mg/l)** | **Initial OD** | **Final OD** | **Degradation rate (%)** | **Average Degradation rate (%)** | **Duration of observation** |
| --- | --- | --- | --- | --- | --- |
|  | 0.20 | 0.00 | 100 |  |  |
| 15 | 0.20 | 0.00 | 100 | 100 | 72 hours |
|  | 0.20 | 0.00 | 100 |  |  |
|  | 0.22 | 0.05 | 77.27 |  |  |
| 30 | 0.22 | 0.05 | 77.27 | 77.27 | 72 hours |
|  | 0.22 | 0.05 | 77.27 |  |  |
|  | 0.24 | 0.09 | 62.5 |  |  |
| 50 | 0.24 | 0.09 | 62.5 | 62.5 | 72 hours |
|  | 0.24 | 0.09 | 62.5 |  |  |

**
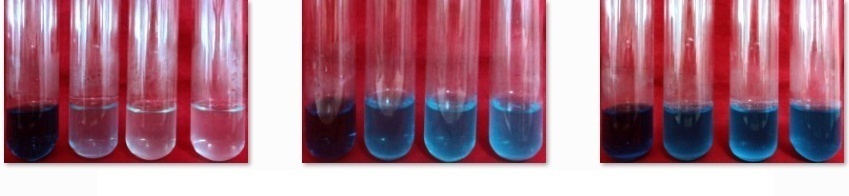
**

**Control**

**Control**

**Control**

**A**

**B**

**C**

**Figure 4.17A:** Effect of malachite green dye concentration on degradation by *Enterobacter* sp. CV–S1: (**A)** 15 mg/l, (**B)** 30 mg/l, (**C)** 50 mg/l

**Figure 4.17B:** Graphical presentation of the effect of malachite green dye concentration on degradation by *Enterobacter* sp. CV–S1

**Figure 4.17C:** 15 mg/l malachite green dye degradation rate at different time intervals by *Enterobacter* sp. CV–S1 using 5% inoculum

**4.2.6 Mixed (Crystal Violet and Malachite Green) Dye Treatment by *Enterobacter* sp. CV–S1**

As in section 4.11.5 *Enterobacter* sp. CV–S1 (5%) showed complete degradation of 15 mg/l (10 times lower than crystal violet which was 150 mg/l) of malachite green within 72 hours, crystal violet and malachite green were jointly treated. As utilization of malachite green was lower than crystal violet in this section the used proportion of crystal violet and malachite green was 2:1 and inoculum size of *Enterobacter* sp. CV–S1 was 10%. Results on investigation on mixed (crystal violet and malachite green) dye treatment by *Enterobacter* sp. CV–S1 are presented in Figure Table 4.12 and 4.18A–C.

72 hours incubation under shaking condition showed up to 50 + 25 mg/l 100% degradation (Figure 4.18A). More than 50 + 25 mg/l concentration the degradation rate was reduced (Figure 4.18B). Lower than 50 + 25 mg/l concentration 25 +12.5 mg/l was degraded completely within 24 hours. At different time intervals mixed (crystal violet and malachite green) dye degradation rate by *Enterobacter* sp. CV–S1 is shown in Figure 4.18C; which indicates complete degradation of mixed dye of the above concentration occurs within 54-60 hours.

**Table 4.12:** Effect of mixed (crystal violet and malachite green) dye concentration on degradation by *Enterobacter* sp. CV–S1

| **Dye concentration [(*CV + **MG) mg/l]** | **Initial OD** | **Final OD** | **Degradation rate (%)** | **Average Degradation rate (%)** | **Duration of observation** |
| --- | --- | --- | --- | --- | --- |
|  | 0.19 | 0.00 | 100 |  |  |
| 25 + 12.5 | 0.19 | 0.00 | 100 | 100 | 24 hours |
|  | 0.19 | 0.00 | 100 |  |  |
|  | 0.22 | 0.00 | 100 |  |  |
| 50 + 25 | 0.22 | 0.00 | 100 | 100 | 72 hours |
|  | 0.22 | 0.00 | 100 |  |  |
|  | 0.24 | 0.02 | 91.67 |  |  |
| 75 + 37.5 | 0.24 | 0.02 | 91.67 | 91.67 | 72 hours |
|  | 0.24 | 0.02 | 91.67 |  |  |
| *CV – Crystal violet  **MG – Malachite green | |  |  |  |  |


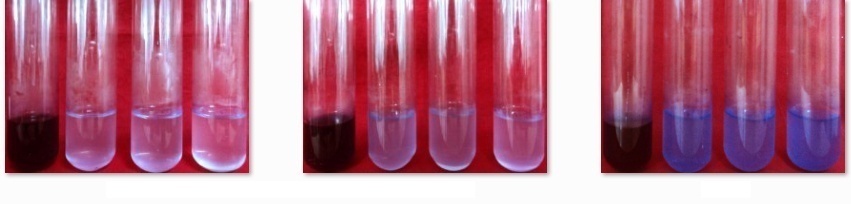


**Control**

**Control**

**Control**

**A**

**B**

**C**

**Figure 4.18A:** Effect of initial mixed (crystal violet and malachite green) dye concentration on degradation by *Enterobacter* sp. CV–S1: (**A**) 37.5 (25 + 12.5) mg/l, (**B)** 75 (50 + 25) mg/l, (**C)** 112.5 (75 + 37.5) mg/l

**Figure 4.18B:** Graphical presentation of the effect of initial mixed (crystal violet and malachite green) dye concentration on degradation by *Enterobacter* sp. CV–S1

**Figure 4.18C:** Mixed (crystal violet and malachite green) dye degradation rate by *Enterobacter* sp. CV–S1 at different time intervals

After optimizing the environmental parameters it was observed that 100% degradation of 150 mg/l crystal violet and 50 + 25 mg/l mixed (crystal violet and malachite green) had occurred within 72 hours at pH 6.50 and 35°C under aerobic shaking condition by 10% (v/v) *Enterobacter* sp. CV–S1 and *Enterobacter* sp. CM–S1 respectively without supplying extra carbon and nitrogen source. Providing optimum conditions 150 mg/l crystal violet and 75 mg/l mixed (crystal violet 50 mg/l and malachite green 25 mg/l) dye degradation at different time intervals are shown in Figure 4.19.

On the other hand, from the mixed dye degradation curve, it was observed that complete degradation of 50 + 25 mg/l (crystal violet and malachite green) dye by *Enterobacter* sp. CV–S1 had occurred around 54–60 hours (Figure 4.18C). This result indicates that *Enterobacter* sp. CV–S1 is more efficient in case for mixed (crystal violet and malachite green) dye degradation than *Enterobacter* sp. CM–S1 though *Enterobacter* sp. CV–S1 was isolated for crystal violet dye treatment. The comparison of mixed (crystal violet and malachite green) dye degradation efficiency by *Enterobacter* sp. CV–S1 and *Enterobacter* sp. CM–S1 are graphically presented in Figure 4.20.

The present findings for crystal violet and malachite green dye degradation individually or jointly by *Enterobacter* sp. CV–S1 and *Enterobacter* sp. CM–S1 is really comparable with the other reports where in maximum cases extra carbon and nitrogen sources were applied for dye degradation (Cheriaa *et al*., 2012, Mukherjee and Das, 2013). There are very few reports on dye degradation using only MS medium. Chen *et al*. (2007) found that 0.022 mg/l of crystal violet decolorization was occurred up to 78.5% by *Pseudomonas putida* within a week at pH 7.50 and 37°C in mineral salt medium where only the dye was the source of carbon and nitrogen.

But, the most optimistic result was observed on decolorization by *Saccharomyces cerevisiae* MTCC 463. The triphenylmethane dye, malachite green (100mg/l) underwent 85% decolorization in plain distilled water within 7 hours (Jadhav and Govindwar, 2006).

**B**

**A**

**Figure 4.19:** Dye degradation rate after optimizing the environmental parameters at different time intervals: **(A)** Crystal violet by *Enterobacter* sp. CV–S1, **(B)** Mixed (crystal violet and malachite green) by *Enterobacter* sp. CM–S1

**Figure 4.20:** The comparison of mixed (crystal violet and malachite green) dye degradation efficiency by *Enterobacter* sp. CV–S1 and *Enterobacter* sp. CM–S1
