# Supplementary material for: Biodegradation of Crystal Violet dye by bacteria isolated from textile industry effluents
Source: PeerJ. 2018 Jun 21;6:e5015. doi: 10.7717/peerj.5015 (PMC6015751; doi:10.7717/peerj.5015)
Supplement: Supplemental Information 7 [file peerj-06-5015-s007.docx]

| **Isolates** | **Initial OD** | **Final OD** | **Degradation rate (%)** | **Average degradation rate (%)** | **Duration of observation** |
| --- | --- | --- | --- | --- | --- |
|  | 0.08 | 0.015 | 81.25 |  |  |
| CV–S1 | 0.08 | 0.015 | 81.25 | 81.25 | 72 hours |
|  | 0.08 | 0.015 | 81.25 |  |  |
|  | 0.08 | 0.03 | 62.50 |  |  |
| CV–S2 | 0.08 | 0.03 | 62.50 | 64.58 | 72 hours |
|  | 0.08 | 0.025 | 68.75 |  |  |
|  | 0.08 | 0.06 | 25.00 |  |  |
| CV–S3 | 0.08 | 0.06 | 25.00 | 25.00 | 72 hours |
|  | 0.08 | 0.06 | 25.00 |  |  |

**Table :** Screening result of dye decolorizing isolates
