# Supplementary figures and images for: Biodegradation of Crystal Violet dye by bacteria isolated from textile industry effluents
Source: PeerJ. 2018 Jun 21;6:e5015. doi: 10.7717/peerj.5015 (PMC6015751; doi:10.7717/peerj.5015)

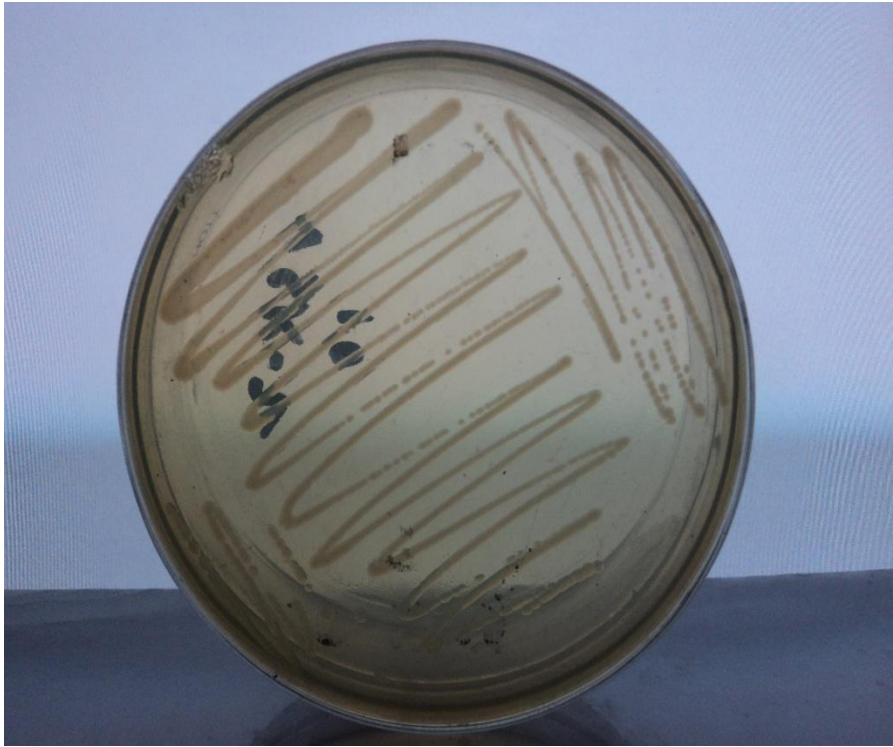

Plate 2: Observation of bacterial growth after 72 hours of incubation with dye solution

Supplement: Supplemental Information 8 [file peerj-06-5015-s008.pdf]
