# Supplementary material for: Biodegradation of Crystal Violet dye by bacteria isolated from textile industry effluents
Source: PeerJ. 2018 Jun 21;6:e5015. doi: 10.7717/peerj.5015 (PMC6015751; doi:10.7717/peerj.5015)
Supplement: Supplemental Information 9 [file peerj-06-5015-s009.docx]

Sequence of CV-S1:

CGTGCATTCTGATCTACGATTACTAGCGATTCCGACTTCATGGAGTCGAGTTGCAGACTCCAATCCGGACTACGACGCACTTTATGAGGTCCGCTTGCTCTCGCGAGGTCGCTTCTCTTTGTATGCGCCATTGTAGCACGTGTGTAGCCCTGGTCGTAAGGGCCATGATGACTTGACGTCATCCCCACCTTCCTCCAGTTTATCACTGGCAGTCTCCTTTGAGTTCCCGGCCTGACCGCTGGCAACAAAGGATAAGGGTTGCGCTCGTTGCGGGACTTAACCCAACATTTCACAACACGAGCTGACGACAGCCATGCAGCACCTGTCTCACAGTTCCCGAAGGCACCAAAGCATCTCTGCTAAGTTCTGTGGATGTCAAGACCAGGTAAGGTTCTTCGCGTTGCATCGAATTAAACCACATGCTCCACCGCTTGTGCGGGCCCCCGTCAATTCATTTGAGTTTTAACCTTGCGGCCGTACTCCCCAGGCGGTCGATTTAACGCGTTAGCTCCGGAAGCCACGCCCTCAAGGGCACAACCTCCCAAATCGACATCGTTTACGGCGTGGAACTACCAAGGTA
